# Supplementary material for: Aging selectively dampens oscillation of lipid abundance in white and brown adipose tissue
Source: Sci Rep. 2021 Mar 15;11:5932. doi: 10.1038/s41598-021-85455-4 (PMC7961067; doi:10.1038/s41598-021-85455-4)
Supplement: Supplementary file 1 — Supplementary Information 1. [file 41598_2021_85455_MOESM1_ESM.pdf]

Aging selectively dampens oscillation of lipid abundance in white and brown adipose tissue

Ntsiki M. Held, Renate Buijink, Hyung L. Elfrink, Sander Kooijman, Georges E. Janssens, Angela C.M. Luyf, Mia L. Pras-Raves, Frédéric M. Vaz, Stephan Michel, Riekelt H. Houtkooper, Michel van Weeghel

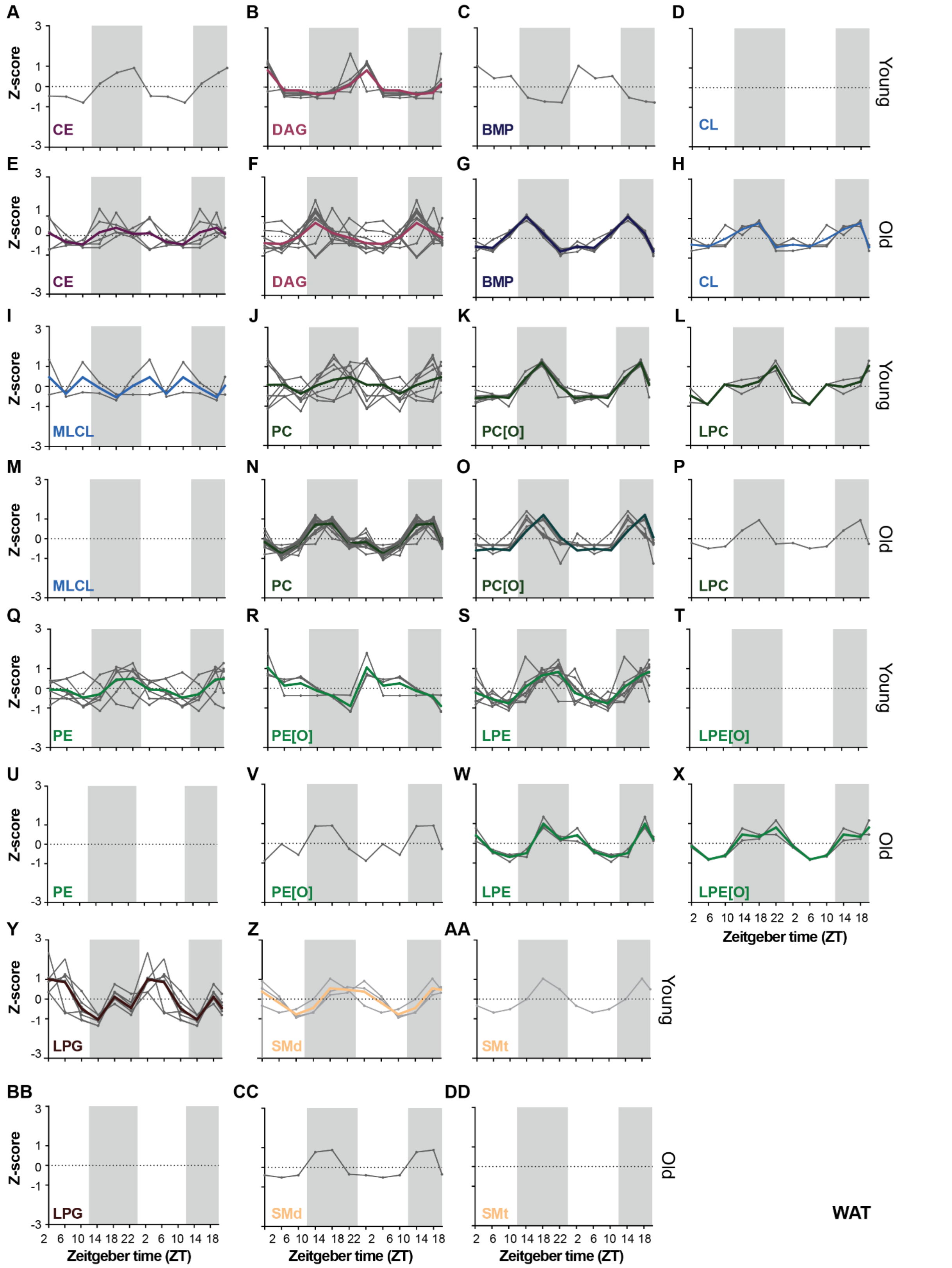

Supplementary Figure S1 Related to Figure 4: Rhythmic lipid profiles in WAT. (**A-D**), (**I-L**), (**Q-T**), (**Y-AA**) lipid profiles of young and (**E-H**), (**M-P**), (**U-X**), (**BB-DD**) old mice. The gray lines are the individual rhythmic lipid species, the bold and colored line represents the average of the rhythmic lipid class. Graphs were created in Graphpad Prism (v8.3.0; https://www.graphpad.com/scientific-software/prism/). Figure layout was prepared in Adobe Illustrator CS6 (v16.0.3; https://www.adobe.com/nl/).

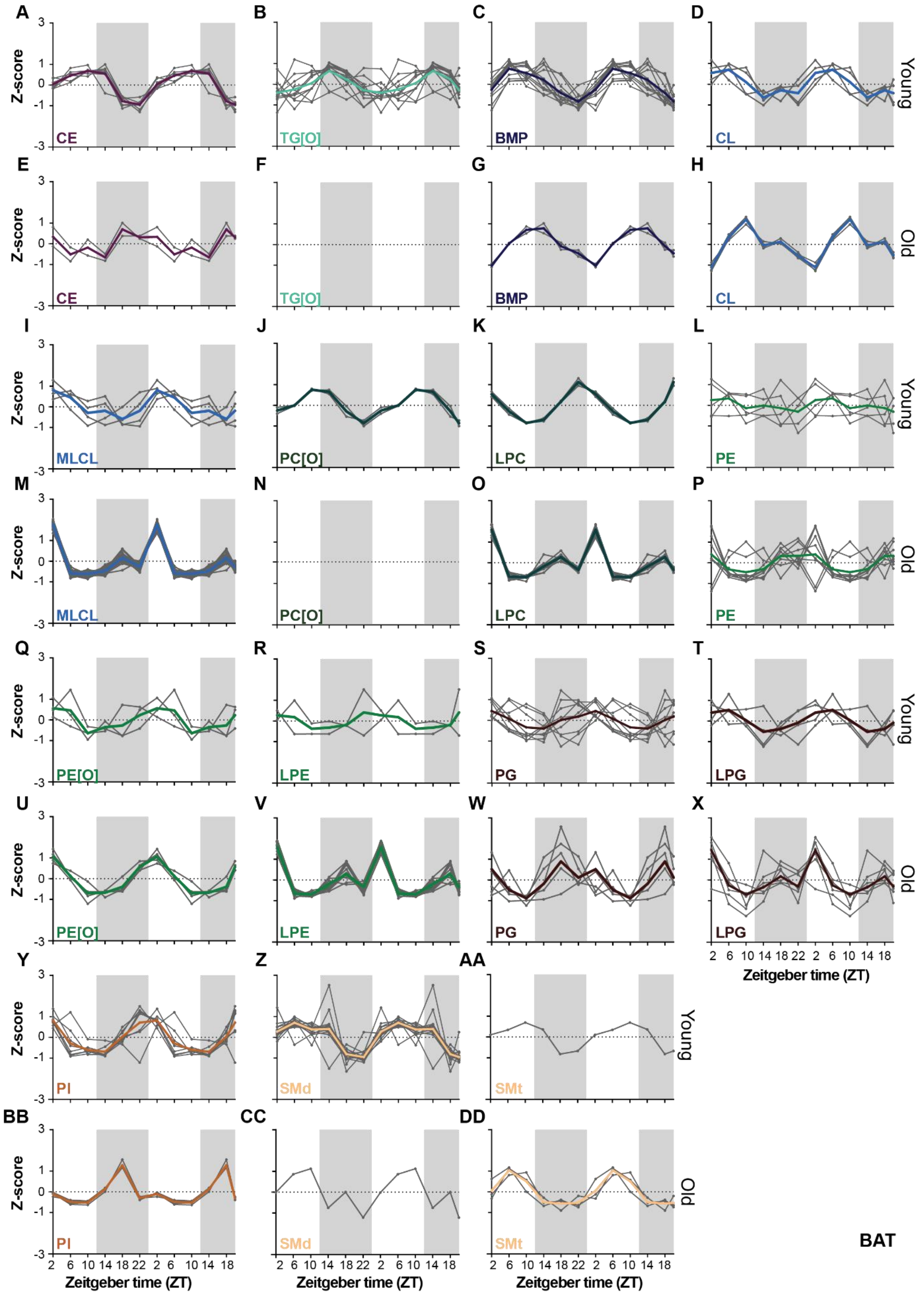

Supplementary Figure S2 Related to Figure 5: Rhythmic lipid profiles in BAT. (**A-D**), (**I-L**), (**Q-T**), (**Y-AA**) lipid profiles of young and (**E-H**), (**M-P**), (**U-X**), (**BB-DD**) old mice. The gray lines are the individual rhythmic lipid species, the bold and colored line represent the average of the rhythmic lipid class. Graphs were created in Graphpad Prism (v8.3.0; https://www.graphpad.com/scientific-software/prism/). Figure layout was prepared in Adobe Illustrator CS6 (v16.0.3; https://www.adobe.com/nl/).
